# WTAP promotes osteosarcoma tumorigenesis by repressing HMBOX1 expression in an m^6^A-dependent manner

**DOI:** 10.1038/s41419-020-02847-6

**Published:** 2020-08-19

**Authors:** Shijie Chen, Yuezhan Li, Shuang Zhi, Zhiyu Ding, Weiguo Wang, Yi Peng, Yan Huang, Ruping Zheng, Haiyang Yu, Jianlong Wang, Minghua Hu, Jinglei Miao, Jinsong Li

**Affiliations:** 1 Department of Orthopaedics, The Third Xiangya Hospital of Central South University, 138 Tongzipo Rd, Changsha, Hunan 410013 China; 2 Shanghai Key Laboratory of Regulatory Biology, Institute of Biomedical Sciences and School of Life Sciences, East China Normal University, Shanghai, 200241 China; 3 Four Gynecological Wards, Ningbo Women and Children’s Hospital, Ningbo, Zhejiang 315000 China; 4 The Second Xiangya Hospital of Central South University, Changsha, China; 5 School of Basic Medical Science, Central South University, Changsha, China; 6 Department of Anatomy, Histology and Embryology, Changsha Medical University, Changsha, China

**Keywords:** Outcomes research, Oncogenesis

## Abstract

N^6^-methyladenosine (m^6^A) regulators are involved in the progression of various cancers via regulating m^6^A modification. However, the potential role and mechanism of the m^6^A modification in osteosarcoma remains obscure. In this study, WTAP was found to be highly expressed in osteosarcoma tissue and it was an independent prognostic factor for overall survival in osteosarcoma. Functionally, WTAP, as an oncogene, was involved in the proliferation and metastasis of osteosarcoma in vitro and vivo. Mechanistically, M^6^A dot blot, RNA-seq and MeRIP-seq, MeRIP-qRT-PCR and luciferase reporter assays showed that HMBOX1 was identified as the target gene of WTAP, which regulated HMBOX1 stability depending on m^6^A modification at the 3′UTR of HMBOX1 mRNA. In addition, HMBOX1 expression was downregulated in osteosarcoma and was an independent prognostic factor for overall survival in osteosarcoma patients. Silenced HMBOX1 evidently attenuated shWTAP-mediated suppression on osteosarcoma growth and metastasis in vivo and vitro. Finally, WTAP/HMBOX1 regulated osteosarcoma growth and metastasis via PI3K/AKT pathway. In conclusion, this study demonstrated the critical role of the WTAP-mediated m^6^A modification in the progression of osteosarcoma, which could provide novel insights into osteosarcoma treatment.

## Introduction

Osteosarcoma is a primary malignant bone tumor that is common among childhood and adolescents worldwide^1^. Despite the improvements including multi-agent chemotherapy with surgery in recent years, the 5-year survival rate is ~70% for localized osteosarcoma and is ~30% for recurrent and metastatic osteosarcoma^2,3^. Therefore, a better understanding of molecular mechanism is urgent for developing novel therapeutic strategies for osteosarcoma.

N6-methyladenosine (m^6^A) is the prominent dynamic mRNA modification, which is involved in various biological process by regulating mRNA translocation, translation, and stability^4^. It is catalyzed by m^6^A writer (methyltransferase), removed by erasers (RNA demethylases) and recognized by m^6^A readers, involving in various biological progression^5–7^. The formation of m^6^A is catalyzed by a methyltransferase, METTL3 and METTL14 form a core catalytic complex of methyltransferases that is stabilized by WTAP^8^. Recently, METTL16^9,10^, METTL5, ZCCHC4^11^, and Zc3h13^12^ were showed to play a critical role in compositing methyltransferase complex and facilitating m^6^A methylation^13^. The eraser ALKBH5 and FTO has m^6^A demethylation activity to remove the m^6^A modification. The reader proteins are from YTH family, heterogeneous nuclear ribonucleoprotein (HNRP) family and insulin-like growth factor 2 mRNA-binding protein family, they recognize the m^6^A modification to adjust RNA metabolisms^14^.

Dynamic and reversible m^6^A regulation was reported to be involved in various physiological and pathological processes including stem cell differentiation, cardiac homeostasis, adipogenesis, neuronal disorders, and spermatogenesis^15–19^. Recent years, compelling evidence has revealed that m^6^A modification plays an important role in the tumorigenesis of various cancers^20–24^. For example, METTL3 was reported to play key role in the progression of colorectal carcinoma^25^, bladder cancer^26,27^, gastric cancer^28^, and pancreatic cancer^29^. YTHDF2 was involved the progression of Acute myeloid leukemia (AML) via regulating hematopoietic stem cells (HSCs) activity^4^. FTO was also reported to play a crucial role in the progression of melanoma^30^, breast cancer^31^, gastric cancer^32^, and intrahepatic cholangiocarcinoma. Nevertheless, the role of m^6^A modification in osteosarcoma is still poorly characterized.

WTAP, a Wilms’ tumor 1 (WT1) associated protein^33^, has been reported to be critical in the biological processes including G2/M transition and pre-mRNA splicing^34–36^. In addition, accumulated studies identified the important role of WTAP in the progression of various cancers^37^. For example, WTAP function as oncogene in cholangiocarcinoma^38^, acute myeloid leukemia^39^, colon cancer^40^, ovarian cancer^41^, and diffuse large B-cell lymphoma^42^. Recent studies reported that WTAP is strictly connected to a functional m^6^A methylation complex^43^. Here, we revealed the increased expression of WTAP in osteosarcoma tissue, which was associated with clinicopathological features and poor prognosis in osteosarcoma patients. WTAP function as an oncogenic gene and it promotes the m^6^A modification and progression of osteosarcoma. Mechanistically, WTAP induced the growth and metastasis of osteosarcoma via downregulating HMBOX1 expression in m^6^A-dependent manner. Further investigations demonstrated that WTAP/HMBOX1 regulated osteosarcoma growth and metastasis via PI3K/AKT pathway. Overall, these results imply that WTAP/HMBOX1 may be an important mechanism of osteosarcoma progression and serve as a novel therapeutically target of osteosarcoma.

## Materials and methods

### Clinical samples and ethics

One-hundred-and-four paired fresh osteosarcoma (40 metastatic OS sample and 64 non-metastatic OS sample) and normal tissues were obtained from patients without radiotherapy and/or chemotherapy at The Third Xiangya Hospital of Central South University and then immediately frozen in −80 °C for RNA and protein extraction, or formalin-fixed and paraffin-embedded for further used. Informed consent was obtained from each patient or their guardians, and the study were approved by the Ethics Committee of The Third Xiangya Hospital of Central South University.

### Cell culture, transfection, and infection

The human hFOB1.19 cells and osteosarcoma cell lines (SJSA-1, MG-63, HOS, U2OS, and 143B) were obtained from the cell bank of the Chinese Academy of Sciences (Shanghai, China). The osteosarcoma cells were cultured in DMEM with 10% FBS (Gibco, Grand Island, NY, USA). The hFOB1.19 cells were cultured in DMEM/F-12 (DF-12) with 10% FBS.

The sequences of shRNA cloned into PLKO.1 vector, and then shWTAP-PLKO.1 or shHMBOX1-PLKO.1 were co-transfected with packing and PAX2 plasmids into 293TF cells. Forty-eight hours after transfection, the lentivirus was collected and infected HOS and U2OS cells for 24 h. The 1 μg/ml puromycin was used for screening infected cells. The primers were listed in Table S1.

### Western blot

The proteins were obtained from cells and tissues being lysed with RIPA buffer and then separated by 10% SDS-PAGE. The anti-WTAP (ab195380, Abcam), anti-HMBOX1 (16123-1-AP, Proteintech), anti-YTHDF2 (ab220163, Abcam), anti-PI3K (ab191606, Abcam), anti-pPI3K (ab182651, Abcam), Akt (9272S, Cell Signaling), p-Akt (Ser473) (4051S, Cell Signaling), and anti-GAPDH (ab181602, Abcam) were used for PVDF membrane incubating overnight at 4 °C. The protein expression was detected by incubating with anti-rabbit secondary antibodies.

### RNA-seq analysis and qRT-PCR

Total RNAs were extracted from osteosarcoma tissue and cells using Trizol (ThermoFisher, USA). For RNA-seq analysis, the library construction and Illumina sequencing using the Illumina TruSeq RNA Sample Prep Kit (San Diego, CA, USA). For qPCR validation, the cDNA was obtained using First Strand cDNA Synthesis Kit (TOYOBO). Finally, the mRNA expression was detected using SYBR GREEN (Bio-Rad, California, USA). All primers were in Table S1.

### RNA m^6^A dot blot assay and RNA m^6^A quantification

Total RNAs were extracted from osteosarcoma cells by TRIzol (ThermoFisher, USA), and then NanoDrop3000 was used for detecting RNA quality. The m^6^A content was detected by EpiQuik m^6^A RNA Methylation Quantification Kit (Colorimetric, Epigentek, USA).

The poly(A) RNAs (600, 300, and 150 ng) were spotted onto a nylon membrane (GE Healthcare) for RNA m^6^A dot blot assay, and then incubated with m^6^A antibody (no. ABE572, Merck Millipore, USA) at 4 °C overnight. The m^6^A dots was analyzed using an imaging system (Bio-Rad, USA).

### MeRIP-seq and MeRIP-qRT-PCR

Total RNAs were extracted from osteosarcoma cells by TRizol. The DNA-free fragmented RNAs were incubated with magnetic Dynabeads bounded anti-m^6^A antibody (Abcam, USA) to enrichment the mRNA with m^6^A. And then, beads were treated with Proteinase K and RNA was extracted for MeRIP-seq or validation by qRT-PCR. The primers are in Table S1.

### Cell proliferation assays

The CCK-8 (Cell Counting Kit-8, C0038, Beyotime Biotechnology, China) was used for cell proliferation ability^44^.

### Colony formation assays

The cells were seeded into 6-well plates with a density 2 × 10^3^ cells per wells and cultured in DMEM medium for 2 weeks. And then, 4% paraformaldehyde (PFA) was used to fix the colonies and crystal violet was used to stain as previous described^45^.

### Wound-healing assay

Osteosarcoma cells were cultured for 48 h to reach 80% confluency, and then a straight artificial wound was scraped with a 200 μl pipette tip. The cell migration ability was measured by photographing the distance at 0 and 24 h^45^.

### Transwell invasion assays

The transwell chamber (BD Science, Bedford, MA, USA) was used for invasion assays as previously described^1^. In brief, 2 × 10^5^ osteosarcoma cells were seeded into the upper chambers and incubated for 24 h. The invasive cells were counted and quantified for cell invasion as previous described^45^.

### Luciferase reporter assays

The 3′UTR of HMBOX1 was cloned into pmiGLO vector (Promega, USA). The putative m^6^A sites bases (A) were mutated into bases (C) in 3′UTR using Site-Directed Mutagenesis Kit (Thermo, USA). The WT and Mut plasmids were transfected in osteosarcoma cells, and then the Dual-Luciferase Assay Kit (Promega) was used for detecting the luciferase activity^44^.

### Animal experiments

Nude mice (4 weeks, male) were used for tumor model. All animal care and handling procedures were approved by the Institutional Animal Care and Use Committee of The Third Xiangya Hospital of Central South University, Changsha, China. For the subcutaneous tumor model, 1 × 10^6^ shNC, shWTAP or shHMBOX1 or shWTAP/shHMBOX1 U2OS cells seeded into mice via subcutaneous injection. Tumor volume and tumor weight were measured to analyze tumor growth as previous described^46^. For orthotopic xenograft tumor model, shNC, shWTAP, shHMBOX1, or shWTAP/shHMBOX1 U2OS cells were labeled with a luminescent dye and GFP, and injected into the cavity of the tibia of mice. Thirty days after injection, tumor growth was detected. For the metastasis model, the cells were injected into the tail vein, and the lung metastasis were detected 30 days after injection using in vivo bioluminescence imaging system.

### Immunohistochemistry

Immunohistochemistry (IHC) analysis was performed using anti-WTAP (ab195380, Abcam), anti-HMBOX1 (16123-1-AP, Proteintech) as pervious described^26^.

### Bioinformatics analysis

The GEO dataset GSE87624 and GSE46705 were downloaded and analyzed by R (version 3.5.3, https://www.r-project.org/). The different expressed gene was calculated with edgeR package and identified by the threshold criteria of log2 Fold-change (FC) ≥ 1.75 and adj.*p* < 0.05. GO and KEGG analysis was performed to investigate the potential role of genes. Protein–protein interaction (PPI) network was analyzed by the STRING database.

### Statistical analysis

The SPSS 22.0 (SPSS, Inc., Chicago, IL, USA) was used for the statistical analyses, using ANOVA or Student’s *t*-test in this study. Kaplan–Meier analysis was used for survival. The correlation between WTAP and HMBOX1 were analyzed using Pearson analysis. Statistical significance was defined *p* < 0.05.

## Results

### Elevated WTAP is associated with poor prognosis of osteosarcoma patients

**Fig. 1:**
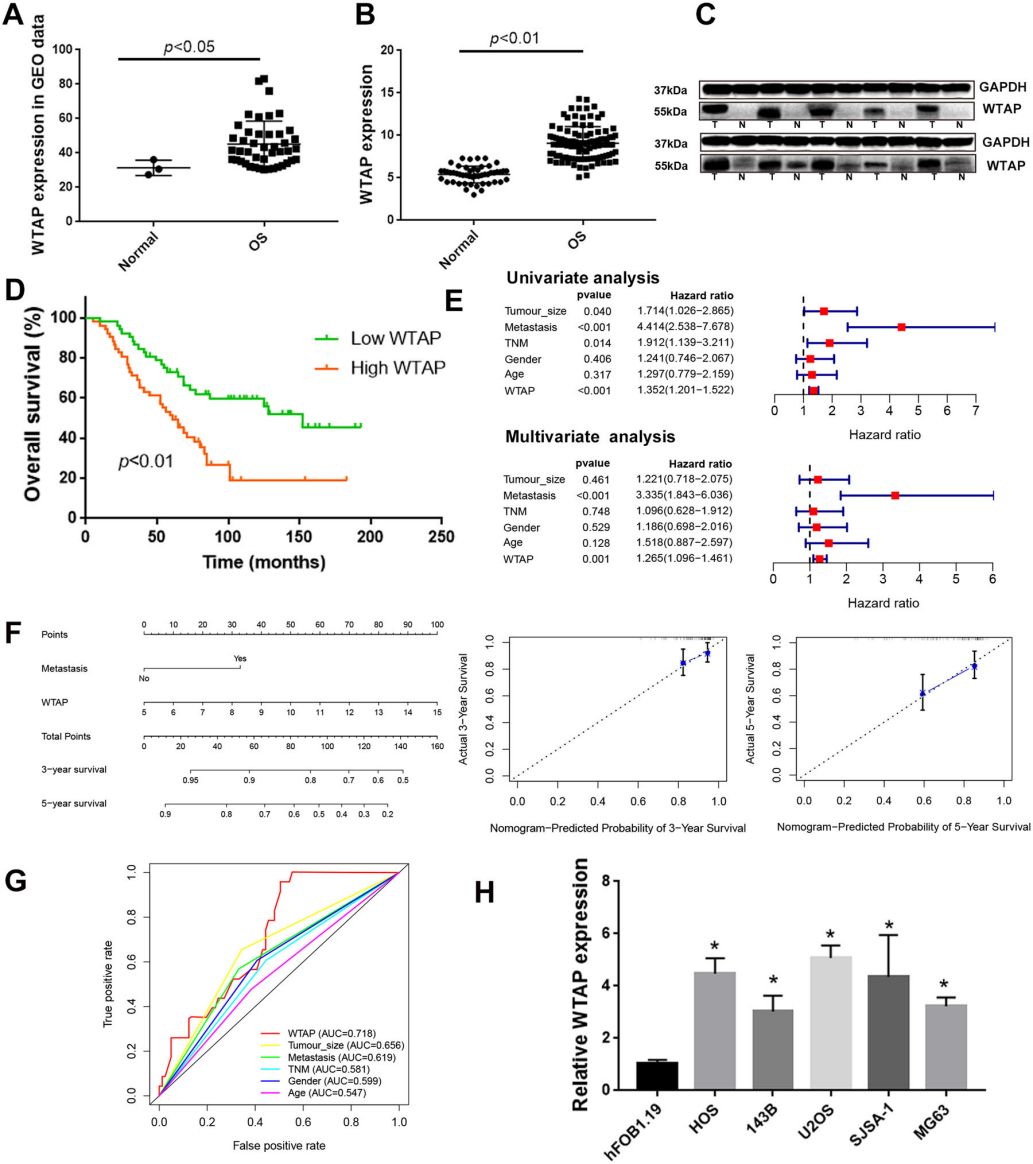
The expression levels of WTAP in osteosarcoma tissue and cell lines. **a** WTAP expression in osteosarcoma tissue compared with normal bone tissue from GSE87624. **b** qPCR assay and **c** western blot assay revealed the WTAP expression in osteosarcoma tissue and the corresponding para-tumor tissues from TXHCSU. **d** The Kaplan–Meier survival analysis. **e** Univariate and multivariate survival analysis. **f** Establishment of the overall survival nomogram for osteosarcoma patients. **g** Time-dependent ROC analysis. **h** The mRNA expression levels of WTAP in osteosarcoma cell lines and hFOB1.19 cell line using qPCR.

To reveal the important role of m^6^A modification in osteosarcoma, we explored the expression levels of 17 m^6^A-relative genes in osteosarcoma tissue and normal bone tissue (GSE87624: 3 normal and 44 osteosarcoma). As shown in Figs. 1a and S1A, we examined the expression of 17 m^6^A-related genes in GEO database, and found that WTAP, RBM15, YTHDF1, and YTHDF2 were differently expressed in tumor tissue compared with control tissue. The m^6^A writers were reported to play key role on the progression of various cancers, so WTAP was selected for further analysis. Next, we detected the expression of WTAP in 104 pairs of osteosarcoma tissue and the corresponding para-tumor tissues from The Third Xiangya Hospital of Central South University (TXHCSU). Consistent with the GEO results, the significant higher mRNA and protein levels of WTAP in osteosarcoma was detected using the qPCR and WB analysis (Fig. 1b, c). Moreover, the osteosarcoma patients with high WTAP showed poor overall survival in TXHCSU (Fig. 1d). And the univariate and multivariate cox analysis showed that WTAP and metastasis were the independent prognostic factor for overall survival in osteosarcoma patients (Fig. 1e). The prognostic nomogram was used to predict the probabilities of overall survival rates of osteosarcoma patients, and the calibration curves showed has a good consistency between the prediction and the actual observation (Fig. 1f). In addition, the 3 years ROC curve (AUC) of WTAP in osteosarcoma patients from TXHCSU was 0.718 (Fig. 1g). Besides, the overexpression of WTAP was associated with tumor size, metastasis, and TNM stage (Table 1 and Fig. S1B). Finally, the higher expression of WTAP was also detected in osteosarcoma cell lines (SJSA-1, MG-63, HOS, U2OS, and 143B) compared with that in the human osteoblast (hFOB1.19) cell (Fig. 1h). Collectively, these results demonstrated that WTAP is highly expressed in osteosarcoma and is correlated with its poor prognosis.

**Table 1:** The association of WTAP expression and clinicopathologic characteristics of osteosarcoma patients.

Characteristics	Case number	WTAP expression		*P* value
		High (*n* = 52)	Low (*n* = 52)	
Gender				*p* > 0.05
Male	48	21	27	
Female	56	31	25	
Age				*p* > 0.05
≤20	60	29	31	
>20	44	23	21	
Tumor size				*p* < 0.01
≤8 cm	60	17	42	
>8 cm	44	33	10	
Metastasis				*p* < 0.01
Yes	40	30	10	
No	64	22	42	
TNM				*p* = 0.015
I/II	55	21	33	
III/IV	49	31	19	

### Silenced WTAP significantly represses osteosarcoma progression in vitro

**Fig. 2:**
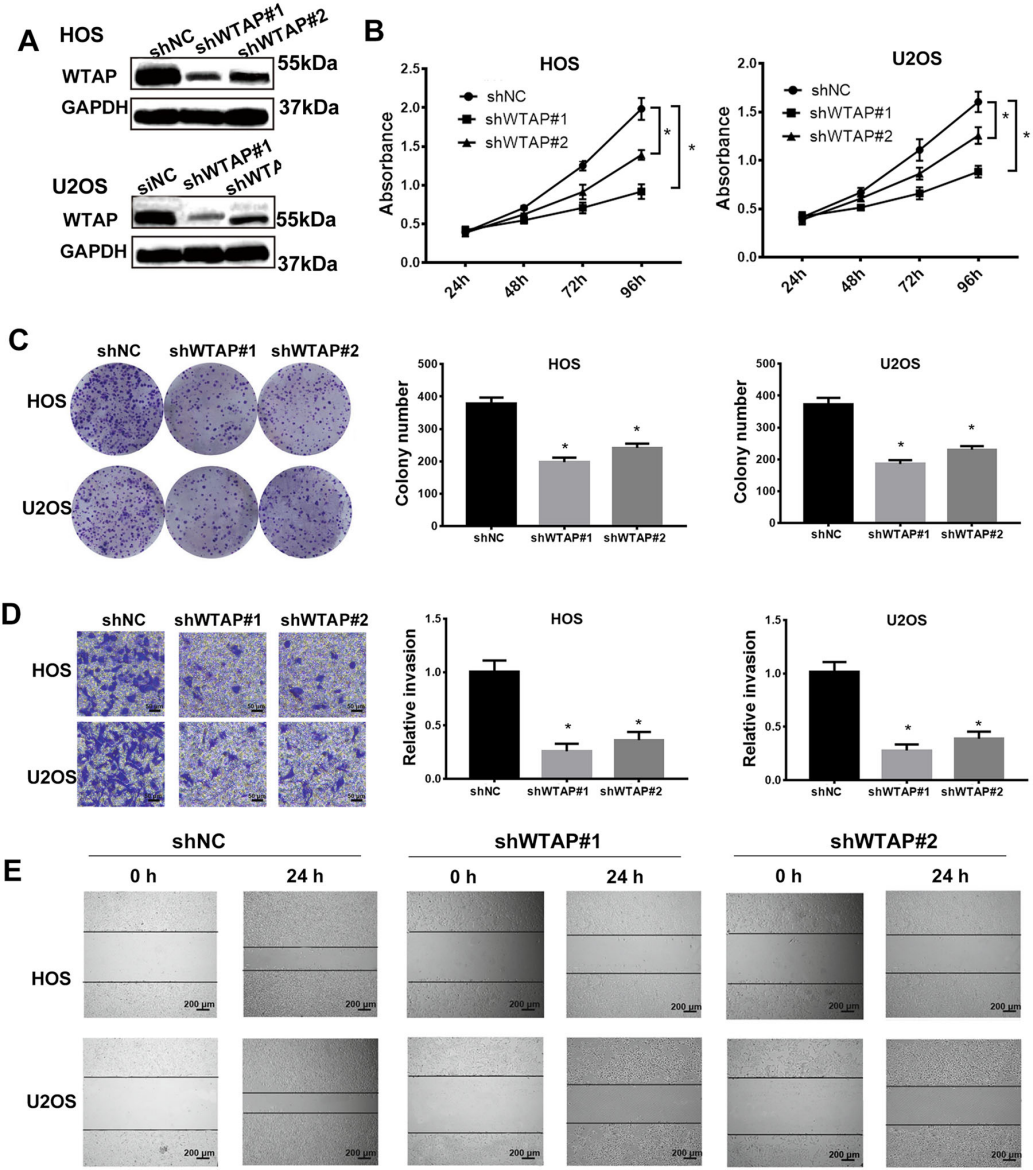
Silenced WTAP significantly repressed osteosarcoma progression in vitro. **a** The shWTAP#1 and shWTAP#2 downregulated WTAP expression. **b** CCK-8 assay revealed that silenced WTAP reduced the cell proliferation ability in osteosarcoma. **c** Colony formation assay showed that silenced WTAP decreased the cloning number of osteosarcoma cells. **d** Transwell invasion and **e** wound-healing assay revealed the inhibition of silenced WTAP on osteosarcoma cell invasion and migration.

To further clarify the role of WTAP in osteosarcoma, we next used shRNA lentivirus (shWTAP#1 and shWTAP#2) to generate stable WTAP-knockdown osteosarcoma cells and analyzed the role of WTAP on cells migration, invasion and proliferation of osteosarcoma. The shWTAP#1 and shWTAP#2 significantly knockdown the expression levels of WTAP in osteosarcoma cells (Fig. 2a). The CCK-8 assay and colony formation assay showed that WTAP deficiency significantly reduced proliferative capacity of osteosarcoma cells (Fig. 2b, c). The transwell invasion and wound-healing assays showed that the invasion and migration ability of the osteosarcoma were significant reduced by silenced WTAP (Fig. 2d, e). These results determine that WTAP acts as an oncogene that promoted cell proliferation, migration, and invasion in osteosarcoma.

### HMBOX1 is potential target of WTAP in osteosarcoma

**Fig. 3:**
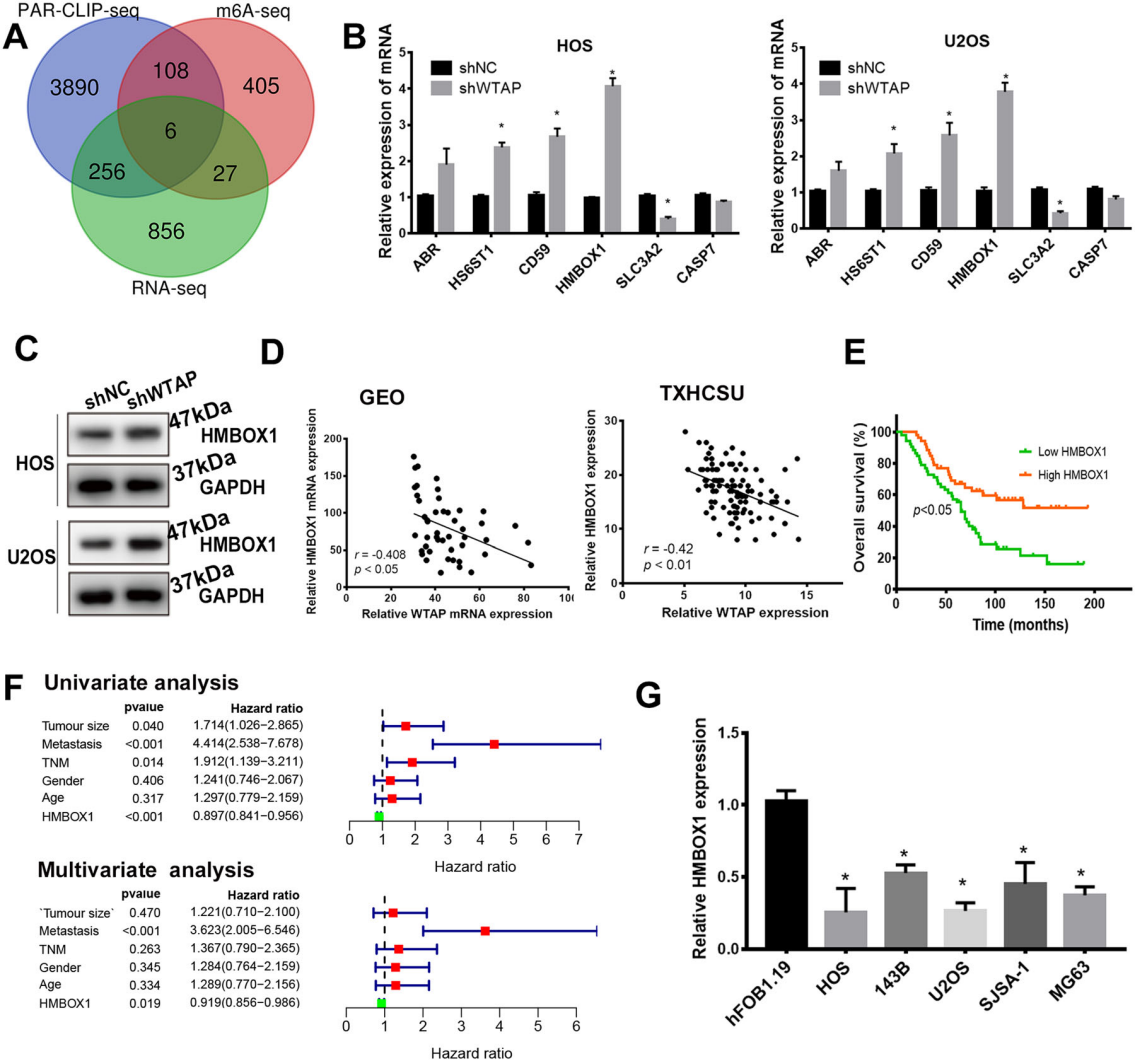
HMBOX1 is a potential target of WTAP. **a** The Venn diagram was generated from differentially expressed genes in RNA-seq, m^6^A-seq, and CLIP-seq in GSE46705. The expression of the overlapped genes in HOS and U2OS cells with silenced WTAP using (**b**) qPCR assay and (**c**) western blot assay. **d** Correlation analysis of WTAP and HMBOX1 in osteosarcoma from GSE87624 and TXHCSU. **e** The Kaplan–Meier survival analysis. **f** Univariate and multivariate survival analyses. **g** WTAP HMBOX1 expression in osteosarcoma cell lines and human osteoblast (hFOB1.19) cell line.

To reveal the potential mechanism by which WTAP regulates the progression of osteosarcoma, we performed RNA-seq, m^6^A-seq in shWTAP/sh-NC U2OS cell and CLIP-seq to reveal the potential genes regulated by WTAP-mediated m^6^A modification. The RNA-seq results revealed 521 downregulated DEGs and 624 upregulated DEGs with |logFC| > 1.75 and adj.*p* < 0.05 in shWTAP group compared with sh-NC group (Fig. S2A). Gene ontology (GO) showed that DEGs were enriched in neutrophil activation, cell cycle, and so on (Fig. S2B). KEGG analysis showed that DEGs were enriched in metabolism-related signal pathway (Fig. S2C). The m^6^A-seq analysis identified 546 differentially m^6^A modification genes in WTAP-silenced U2OS cell compared with normal U2OS cells (Table S2). The CLIP-seq (from GSE46705) revealed 4260 WTAP-targeted RNA in WTAP overexpressed cell using PAR-CLIP technology. And then, we obtained the overlapped genes in RNA-seq, m^6^A-seq, and CLIP-seq as shown in Fig. 3a, 6 genes were overlapped in three groups, including two upregulated gene (SLC3A2, CASP7) and four downregulated gene (ABR, HS6ST1, CD59, and HMBOX1). Consistent with these results, SLC3A2 and CASP7 were significantly upregulated and ABR, HS6ST1, CD59, and HMBOX1 were significantly downregulated in osteosarcoma tissues from GEO (GSE87624) database (Fig. S3A). These results were also confirmed in the osteosarcoma tissues from our TXHCSU data (Fig. S3B). And then, we evaluated the regulation of WTAP on the six candidates in osteosarcoma cells using qPCR. Among which, HMBOX1 was the most significantly upregulated gene in shWTAP osteosarcoma cells (Fig. 3b) and was selected for further analysis. Consistent with the mRNA expression, the protein level of HMBOX1 was also remarkably increased by silenced WTAP (Fig. 3c). We next evaluated the relationship between WTAP and HMBOX1 expression in GSE87624 and TXHCSU. As our speculation, HMBOX1 expression was negatively correlated with WTAP expression (*r* = −0.408 in GSE87624 and *r* = −0.42 in TXHCSU) (Fig. 3d). Moreover, we analyzed the relationship between HMBOX1 expression clinicopathological features in 104 osteosarcoma patients from TXHCSU. HMBOX1 expression was significantly reduced with tumor size and metastasis (Table 2 and Fig. S3C). And the survival analysis results showed that the patients with low level HMBOX1 had poor overall survival in osteosarcoma (Fig. 3e). Moreover, the univariate and multivariate analysis demonstrated HMBOX1 as an independent prognostic factor for overall survival (*p* = 0.019) in osteosarcoma patients (Fig. 3f). We also revealed the lower expression of HMBOX1 in osteosarcoma cell lines (SJSA-1, MG-63, HOS, U2OS, and 143B) than that in the human osteoblast (hFOB1.19) cell line (Fig. 3g). In conclusion, these results indicated that HMBOX1 is a potential target of WTAP and is related to poor prognosis of osteosarcoma patients.

**Table 2:** The association of HMBOX1 expression and clinicopathologic characteristics of osteosarcoma patients.

Characteristics	Case number	HMBOX1 expression		*P* value
		High (*n* = 52)	Low (*n* = 52)	
Gender				*p* > 0.05
Male	48	25	23	
Female	56	27	29	
Age				*p* > 0.05
≤20	60	27	33	
>20	44	25	19	
Tumor size			*p* > 0.05	
≤8 cm	60	34	26	
>8 cm	44	18	26	
Metastasis			*p* < 0.01	
Yes	40	11	29	
No	64	41	23	
TNM				*p* > 0.05
I/II	55	29	26	
III/IV	49	23	26	

### WTAP regulates HMBOX1 expression via m^6^A modification in osteosarcoma

**Fig. 4:**
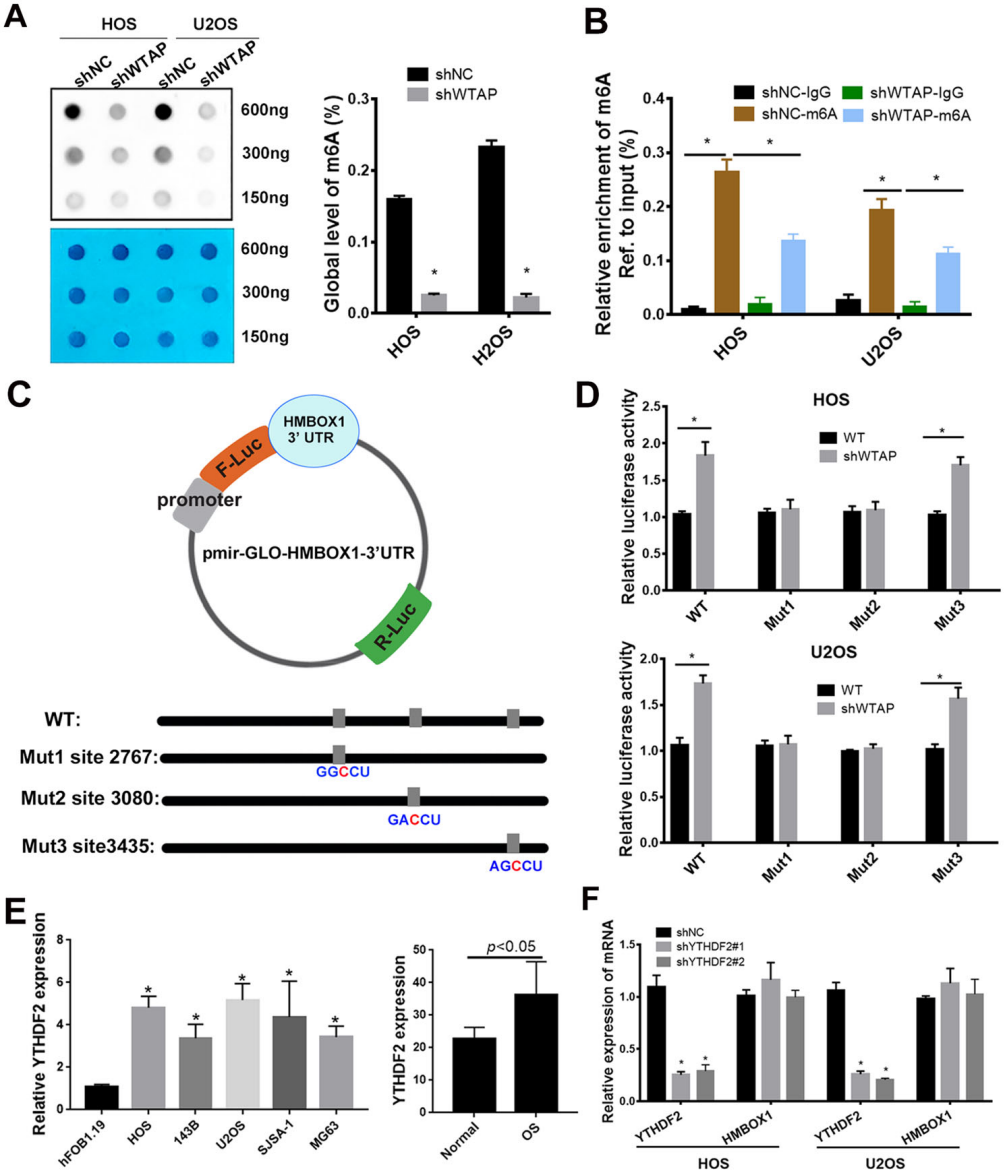
HMBOX1 is negative correlation WTAP expression and is associated to the poor prognosis of osteosarcoma. **a** The m^6^A level in HOS and U2OS cells with silenced WTAP. **b** MeRIP-qPCR assay followed by qRT-PCR revealed the HMBOX1 m^6^A modification. **c** Wild-type or m^6^A site mutant HMBOX1 were cloned in pmirGLO. **d** Luciferase reporter assays revealed the target role of WTAP on the 3′UTR of HMBOX1. **e** YTHDF2 expression in osteosarcoma tissue and cells. **f** The effects of silenced YTHDF2 on HMBOX1 expression in osteosarcoma cells.

As MeRIP-seq data revealed different m^6^A modification of HMBOX1 in NC and WTAP-silenced cells, we next analyzed whether WTAP regulated the HMBOX1 expression in an m^6^A-dependent manner using m^6^A dot blot and RNA methylation quantification assay. As expected, m^6^A levels were substantially decreased in WTAP-knockdown osteosarcoma cells compared control osteosarcoma cells (Fig. 4a). Moreover, MeRIP-qPCR assay showed that HMBOX1 was effectively enriched by m^6^A-specific antibody, and enriched HMBOX1 was remarkably decreased by in WTAP-knockdown cells (Fig. 4b). Therefore, we supposed that WTAP could regulate m^6^A levels of HMBOX1. According to the m^6^A RNA-seq result, the m^6^A modification was at the 3′UTR of HMBOX1, and the SRAMP (http://www.cuilab.cn/sramp) predicted three very high confidence m^6^A sites at the 3′UTR of HMBOX1 (Fig. S4). To further prove the directed target role of WTAP on HMBOX1 with m^6^A modification, we cloned the HMBOX1 3′UTR containing these 3 m^6^A sites into pmirGLO vectors, and then we mutant the bases (A) into bases (C) in the predicted m^6^A sites (Fig. 3f). In Fig. 3g, the luciferase activity of HMBOX1 was significantly increased by silenced WTAP, however, the luciferase activity of Mut HMBOX1 did not affected by silenced WTAP in both HOS and U2OS cells (Fig. 4c).

YTHDF2 is a well know m^6^A reader and plays an important role in the progression of several cancers via regulating mRNA degradation. Figure 1S showed that YTHDF2 was upregulated in osteosarcoma tissues in GSE87624. In Fig. 4e, YTHDF2 evidently upregulated in osteosarcoma tissue and cells. We next shed light on the expression of YTHDF2 in osteosarcoma and the role of YTHDF2 on HMBOX1 expression in osteosarcoma. Disappointedly, silenced YTHDF2 showed no effects on HMBOX1 expression in osteosarcoma cells (Fig. 4f), suggesting that WTAP regulated m^6^A-mediated HMBOX1 expression in YTHDF2-independent manner.

Together, these data suggested that WTAP-repressed HMBOX1 expression via regulating m^6^A modification of HMBOX1 at its 3′UTR.

### HMBOX1 is involved in WTAP-mediated osteosarcoma proliferation and metastasis in vitro

**Fig. 5:**
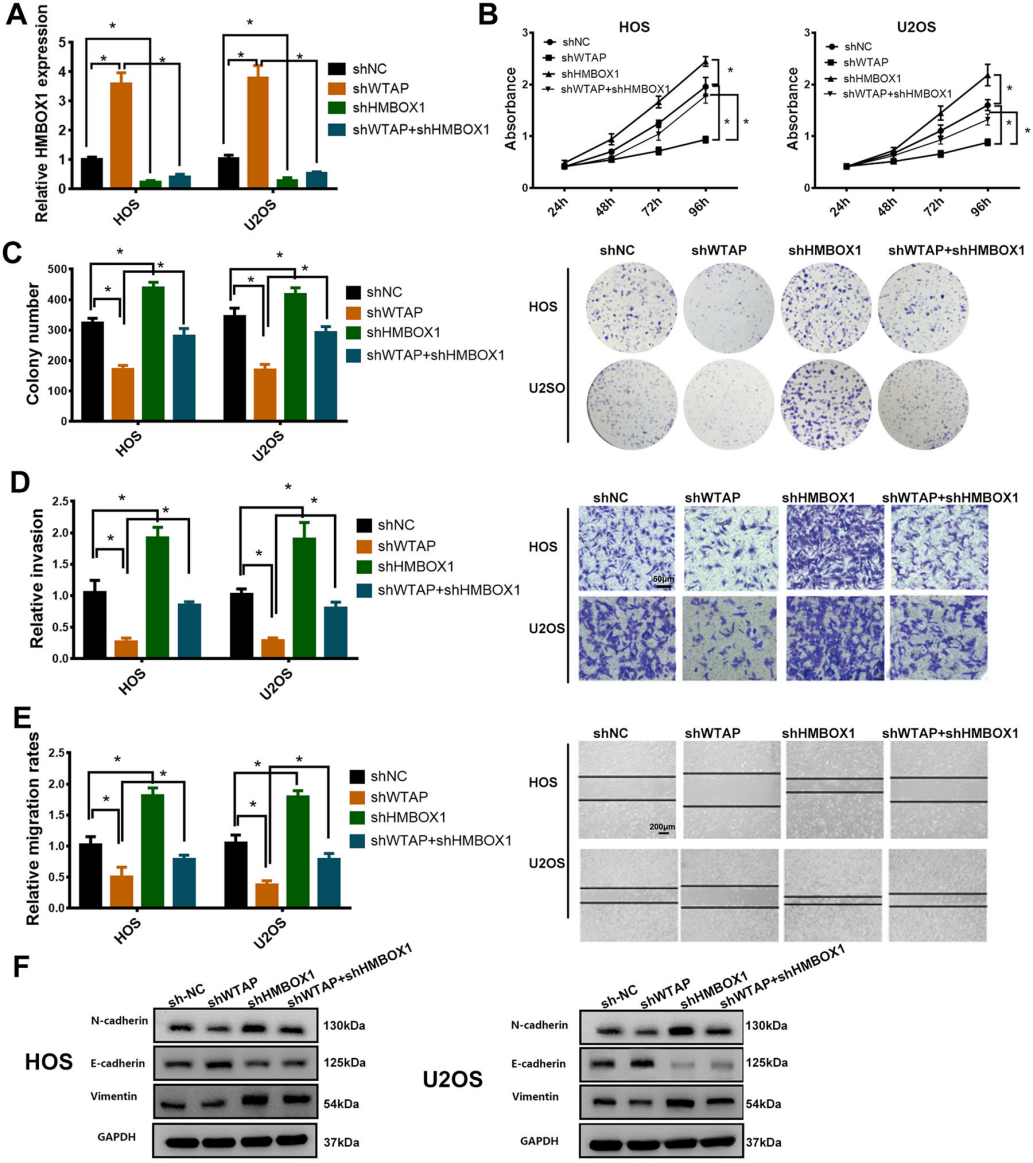
HMBOX1 as a tumor suppressor was involved in WTAP-mediated progression. **a** HMBOX1 expression in osteosarcoma cell with shWTAP and shHMBOX1. Knockdown of HMBOX1 effectively alleviated WTAP-dependent **b** cell proliferation, **c** colony formation, **d** transwell invasion, and **e** wound-healing assay. **f** The protein expression level of EMT transition related protein. *p < 0.05.

We next explored whether WTAP promoted osteosarcoma progression by regulating HMBOX1 expression. As shown in Fig. 5a, HMBOX1 expression was evidently increased by WTAP knockdown and was decreased by HMBOX1 knockdown in osteosarcoma cells. The CCK-8 results showed that the proliferation levels were increased by shHMBOX1 in WTAP-silenced HOS and U2OS cells (Fig. 5b). Consistent with the CCK-8 results, silenced HMBOX1 also alleviated shWTAP-mediated repression of cell proliferation in colony formation assay (Fig. 5c). The similar effects of HMBOX1 were also observed in WTAP-silenced osteosarcoma cell using wound-healing and transwell invasion assays (Fig. 5d and e). In addition, western blot results showed that silenced WTAP evidently repressed the expression of mesenchymal markers (cadherin and vimentin) and induced the expression of epithelial marker E-cadherin which was attenuated by shHMBOX1 in osteosarcoma cells (Fig. 5f). These data suggested WTAP promotes osteosarcoma cells proliferation and metastasis via repressing HMBOX1 expression.

### HMBOX1 inhibits osteosarcoma growth and metastasis in vivo

**Fig. 6:**
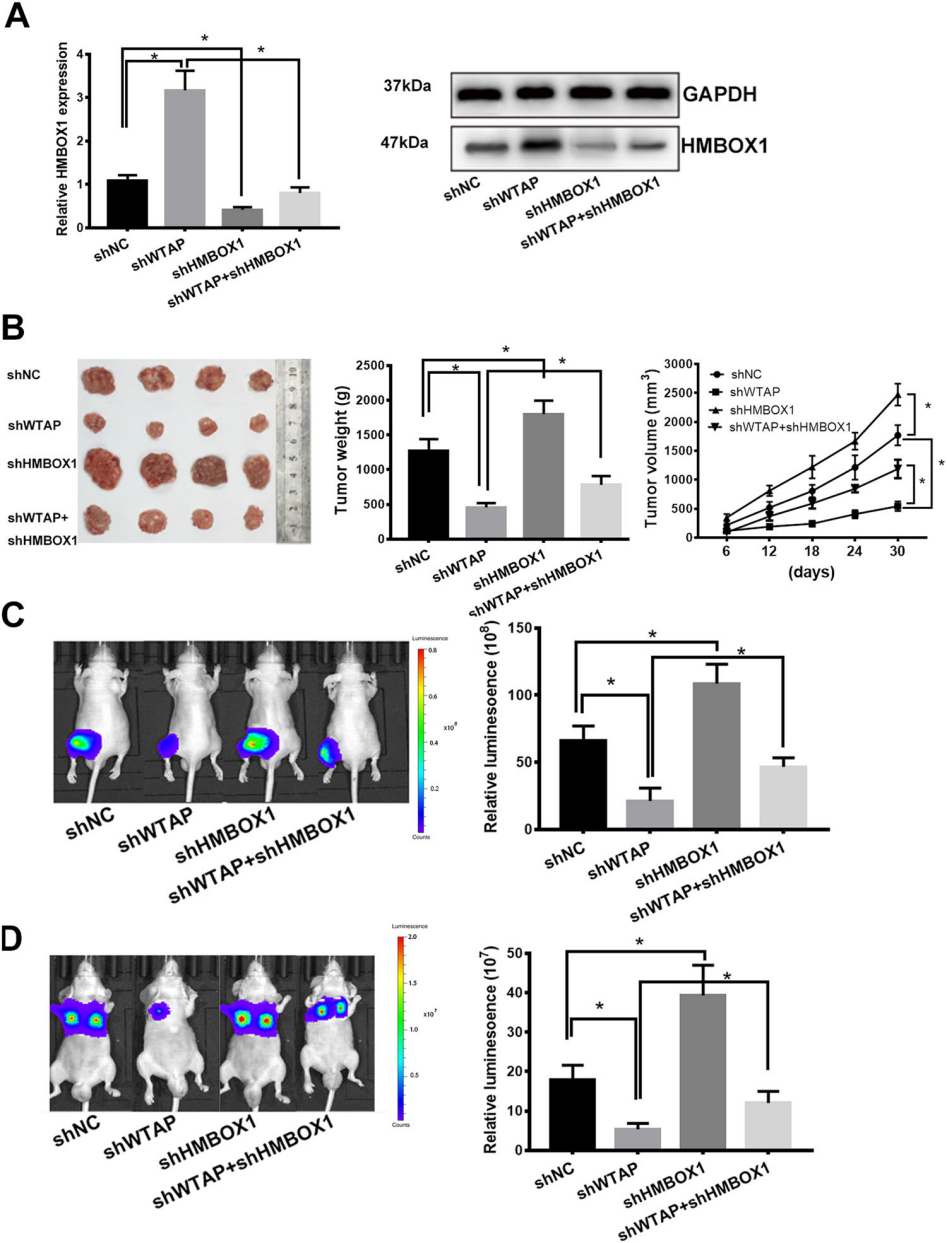
silenced HMBOX1 attenuated shWTAP-repressed osteosarcoma growth and metastasis in vivo. **a** The expression levels of HMBOX1 in osteosarcoma mice models. **b** Knockdown of HMBOX1 effectively alleviated shWTAP-repressed osteosarcoma growth in mice. **c** The orthotopic xenograft tumor and **d** lung metastasis were detected using a vivo bioluminescence imaging system. Representative images and a histogram are shown (*n* = 8 each group).

We next verified the role of HMBOX1 in vivo by injecting shNC, shWTAP, shHMBOX1, and shWTAP/shHMBOX1 U2OS cells to induce subcutaneous osteosarcoma mice model, orthotopic xenograft tumor model, and tail vein metastasis model. The expression levels of HMBOX1 was significantly upregulated by silenced WTAP in subcutaneous osteosarcoma tissue (Figs. 6a and S5 and S6). Moreover, silenced WTAP repressed the tumor size and tumor weight in subcutaneous osteosarcoma mice, which was rescued by silenced HMBOX1 (Fig. 6b). We next used a luminescent dye and GFP labeled U2OS cells to build an orthotopic xenograft tumor model. The bioluminescence imaging showed that the WTAP knockdown reduced the proliferation of U2OS cells in situ, while silenced HMBOX1 alleviated this repression (Fig. 6c). To further detect the role of WTAP and HMBOX1 on the metastatic ability of osteosarcoma in vivo, U2OS cells were injected into nude mice via the tail vein. The bioluminescence imaging showed that silenced HMBOX1 alleviated the repression of silenced WTAP on osteosarcoma metastasis (Fig. 6d). Taken together, these results suggest that HMBOX1 is involved in WTAP-mediated tumor growth and metastasis of osteosarcoma.

### WTAP/HMBOX1 regulates the proliferation and metastasis of osteosarcoma partly by PI3K/AKT pathway

**Fig. 7:**
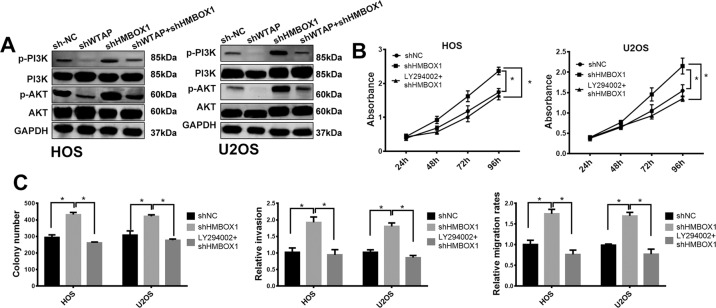
WTAP/ HMBOX1 was involved the progression of osteosarcoma via PI3K/AKT pathway. **a** Western blot analysis for the expression of pPI3K, PI3K, pAKT, and AKT in osteosarcoma cells. **b** Cell proliferation, **c** colony formation, transwell invasion, and wound-healing assay of osteosarcoma cell treated with 5 μM Akt inhibitor LY294002 (Abcam, ab120243) for 12 h.

**Fig. 8:**
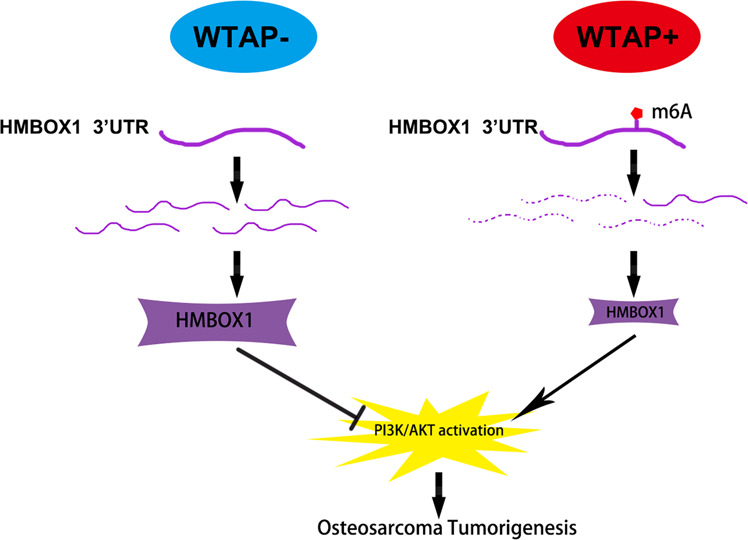
A schematic model illustrating WTAP-mediated m^6^A regulation in osteosarcoma.

The KEGG results identified that PI3K/AKT pathways could be regulated by WTAP (Fig. S2B). PI3K/AKT signaling pathway promotes the growth, migration, and invasion of various cancers including osteosarcoma^47,48^. We hypothesized that WTAP/HMBOX1 was involved in the progression of osteosarcoma via regulating PI3K/AKT signaling pathway. As shown in Fig. 7a, phospho-PI3K and phospho-AKT were evidently repressed by shWTAP and induced by shHMBOX1, and shHMBOX1 significantly attenuated shWTAP-repressed phospho-PI3K and phospho-AKT in both HOS and U2OS cells. LY294002, a PI3K/AKT pathways inhibitor, remarkably reduced shHMBOX1-induced cell proliferation, migration, and invasion in both HOS and U2OS cells (Fig. 7b, c). Therefore, these results imply that WTAP/HMBOX1 regulates the proliferation and metastasis of osteosarcoma partly via PI3K/AKT pathway (Fig. 8).

## Discussion

In the past several years, m^6^A modification is considered as a pervasive internal modification of mRNA and plays critical roles in the progression of a variety of human diseases including cancers^20^. However, the underlying involvement of m^6^A regulators in osteosarcoma progression is still unclear. In the present study, we focused on the role and underlying mechanism of WTAP and it-mediated m^6^A modification in the progression and metastasis of osteosarcoma. In this study, WTAP was firstly identified to upregulated which was associated with poor prognosis of osteosarcoma. Functionally, WTAP promoted the growth and metastasis of osteosarcoma in vitro and vivo. Mechanistically, HMBOX1 was identified as the target gene of WTAP, and it was regulated by WTAP with m^6^A modification at the 3′UTR. Finally, WTAP/HMBOX1 regulated osteosarcoma growth and metastasis in a PI3K/AKT-dependent pattern.

Actually, WTAP was reported as an oncogene in various cancers^37–39,42^. Recent studies reported that WTAP is strictly connected to a functional m^6^A methylation complex^43^. However, few study demonstrated the important role of WTAP as a m^6^A regulator. Here, we concluded that WTAP is not only upregulated but also plays key role on the m^6^A modification in osteosarcoma. Notably, the aberrant of m^6^A modification is related to various biological processes via regulating mRNA stability, splicing and translation. Next, we shed light on the downstream mRNA that modified by WTAP-mediated m^6^A modification by combining the data from RNA-seq, m^6^A-seq, and CLIP-seq. The results showed that HMBOX1 is a potential target gene of WTAP. Subsequently, MeRIP-qPCR, western blot and luciferase reporter assay results revealed that WTAP repressed HMBOX1 expressed with WTAP-dependent m^6^A modification at the 3′UTR of HMBOX1. Thus, WTAP involved in tumorigenesis depending on its role of m^6^A modification. Although YTHDF2 is a well-known m^6^A reader in several cancers. We found that YTHDF2 showed no effects on HMBOX1 expression, suggesting that WTAP regulated m^6^A-mediated HMBOX1 expression in YTHDF2-independent manner. And the m^6^A reader which was involved in the m^6^A modification of HMBOX1 need be further investigated.

HMBOX1, a homeobox containing protein^49^, was reported to be a transcriptional repressor, involving in the biological processes in bone marrow-derived stroma cells^50^, NK cells^51,52^, and vascular endothelial cells^53^. Recent studies demonstrated the dysregulated HMBOX1 in various cancers. For example, high expression of HMBOX1 was observed in gastric cancer, and it was associated with the poor prognosis of gastric cancer^54^. Moreover, HMBOX1 also observed as tumor suppressor in ovarian cancer^55^ and cervical cancer^56^. HMBOX1 repressed the progression of ovarian cancer via regulating cell proliferation and apoptosis^55^. HMBOX1 repressed liver cancer progression via regulating autophagy as well as and immune escape^57^. Consistently, HMBOX1 is downregulated and closely related to the poor prognosis of osteosarcoma in the present study. In addition, silenced HMBOX1 evidently alleviated WTAP-knockdown-mediated repression of osteosarcoma progression, which implied the import roles of HMBOX1 in WTAP-driven osteosarcoma development. Finally, we analyzed the downstream pathway of WTAP/HMBOX1 in osteosarcoma. PI3K/AKT pathway was reported to an important role in the progression of various cancers including osteosarcoma^58^. We found that PI3K/AKT pathway were evidently repressed by shWTAP, which was abolished by HMBOX1 knockdown. Inhibiting PI3K/AKT pathways by LY294002 remarkably reduced shHMBOX1-promoted cell proliferation, migration, and invasion. Therefore, these results imply that WTAP/HMBOX1 regulates the proliferation and metastasis of osteosarcoma partly via regulating PI3K/AKT pathway. However, it remains unclear how HMBOX1, as a transcriptional repressor, regulates PI3K/AKT pathway. Nonetheless, silenced HMBOX1 only partly alleviated WTAP-mediated OS progression. It means that there are other potential molecular mechanisms regulated by WTAP-mediated epigenetic modulation in OS.

In summary, we identified the elevated WTAP in osteosarcoma and which is associated with poor clinical outcome and serves as an independent prognostic factor for osteosarcoma patients. WTAP dramatically promoted osteosarcoma proliferation and metastasis via regulating HMBOX1 mRNA stability in a m^6^A-dependent manner. WTAP/HMBOX1 regulated osteosarcoma growth and metastasis via PI3K/AKT pathway. Altogether, our results determined WTAP/ HMBOX1 as a potential therapeutic target for osteosarcoma.

## Supplementary information

Figure S1

Figure S2

Figure S3

Figure S4

Figure S5

Figure S6

supplementary Figure Legends

SUPPLEMENTAL table 2
