# Supplementary figures and images for: WTAP promotes osteosarcoma tumorigenesis by repressing HMBOX1 expression in an m6A-dependent manner
Source: Cell Death Dis. 2020 Aug 19;11(8):659. doi: 10.1038/s41419-020-02847-6 (PMC7438489; doi:10.1038/s41419-020-02847-6)

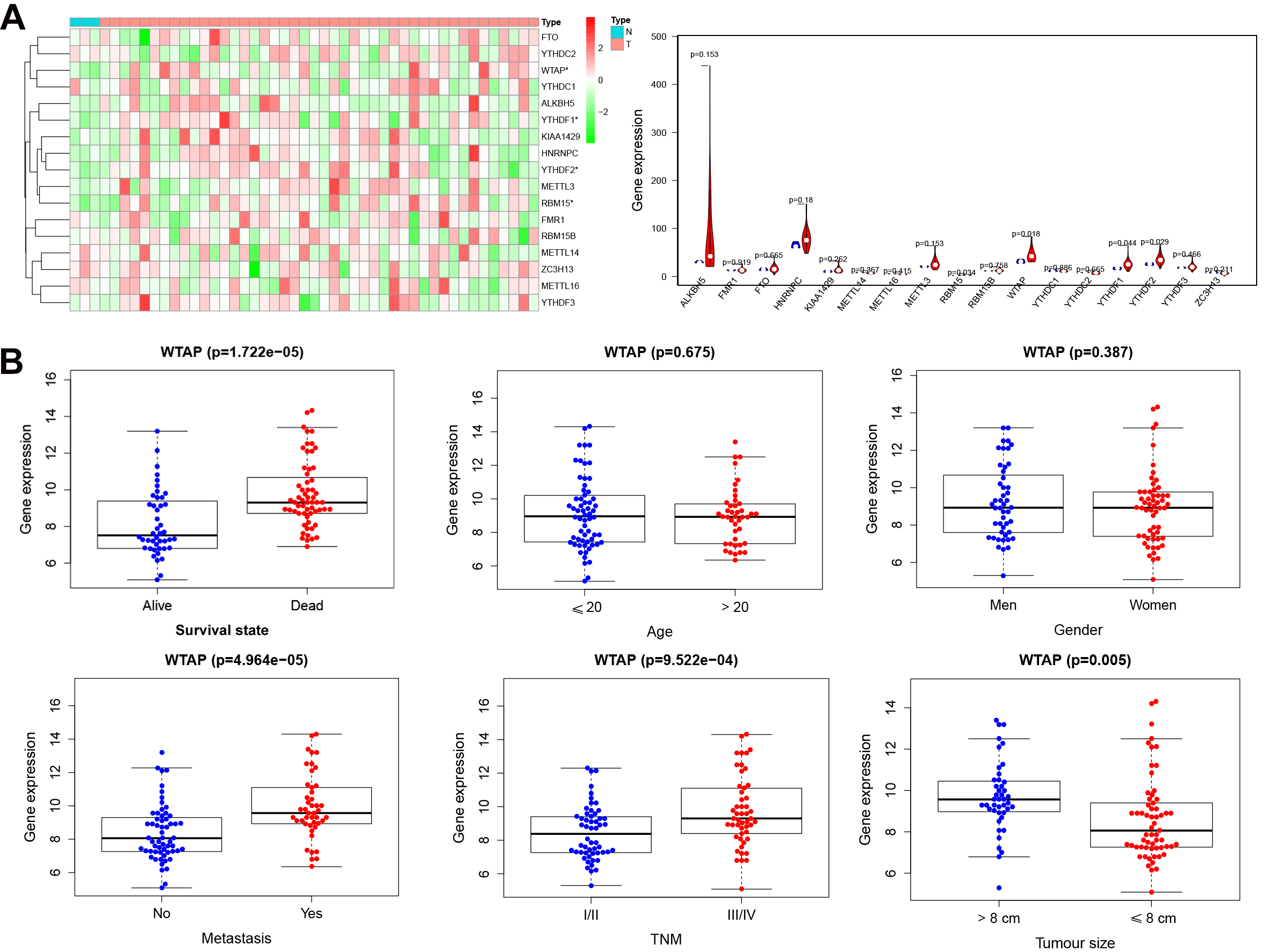

Supplement: Supplementary file 1 — Figure S1 [file 41419_2020_2847_MOESM1_ESM.tif]

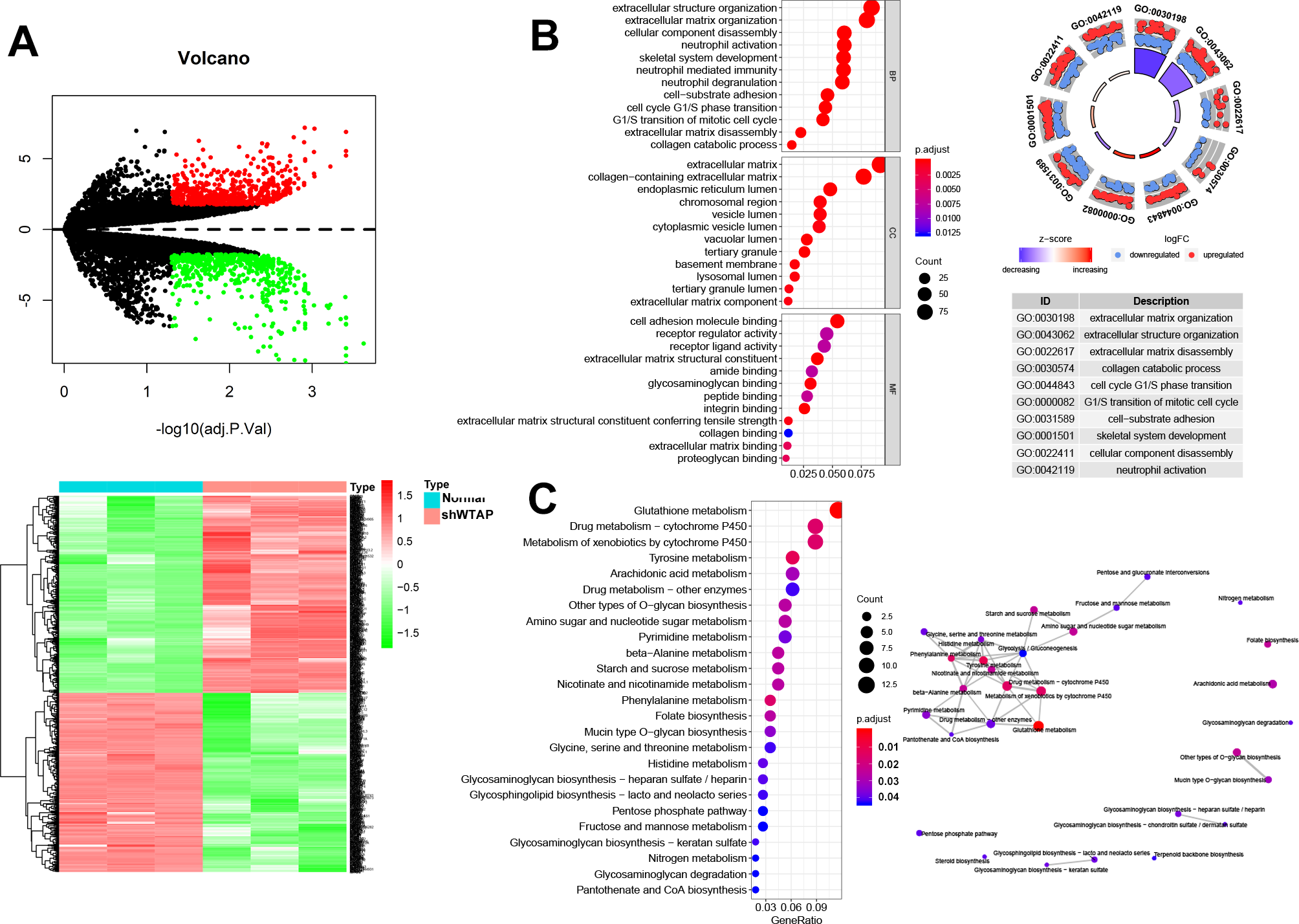

Supplement: Supplementary file 2 — Figure S2 [file 41419_2020_2847_MOESM2_ESM.tif]

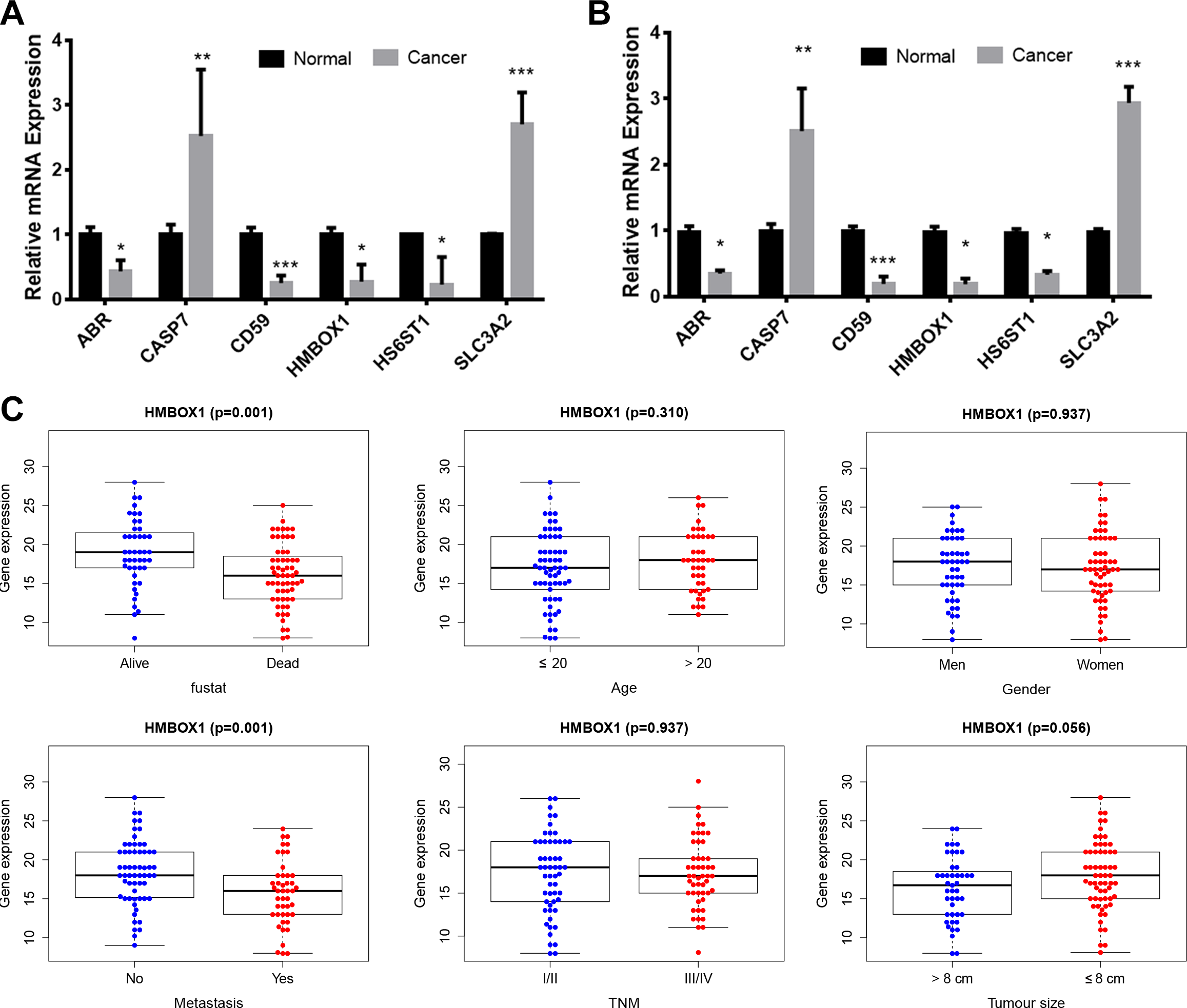

Supplement: Supplementary file 3 — Figure S3 [file 41419_2020_2847_MOESM3_ESM.tif]

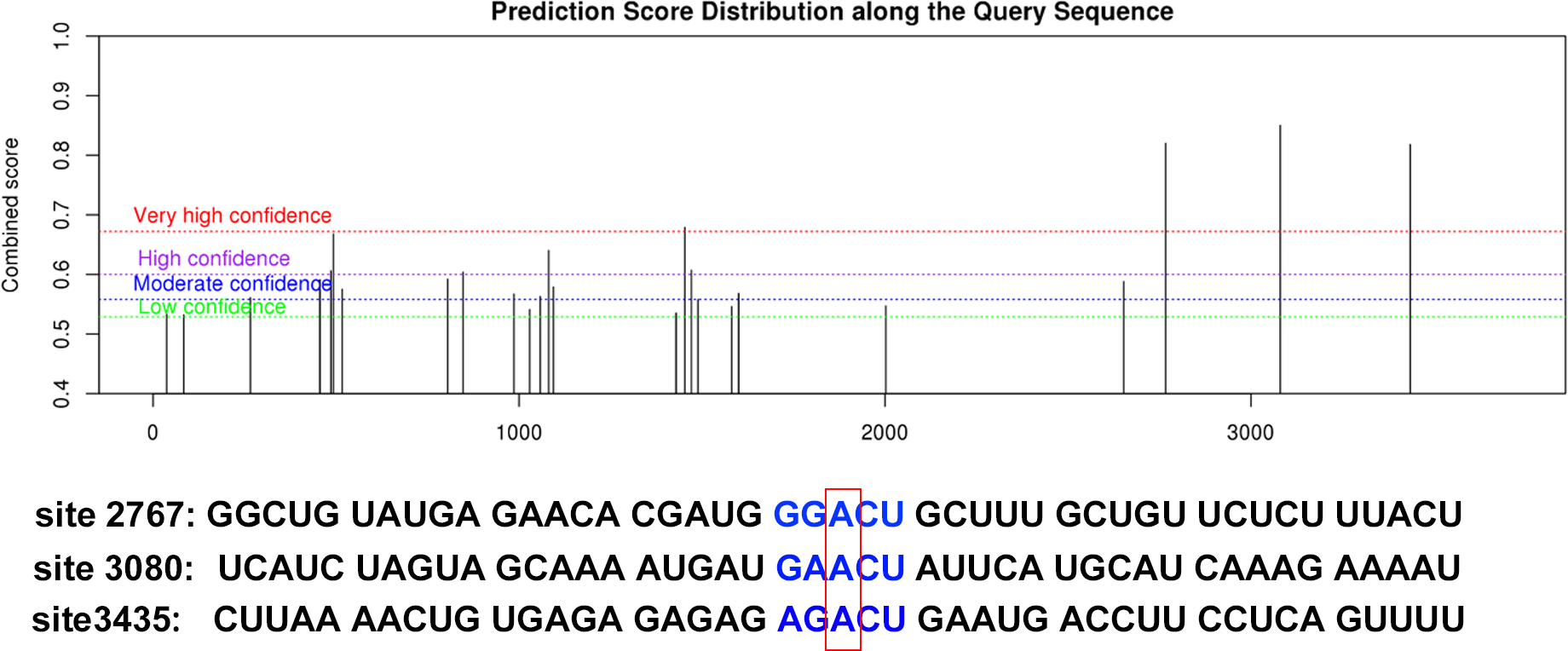

Supplement: Supplementary file 4 — Figure S4 [file 41419_2020_2847_MOESM4_ESM.tif]

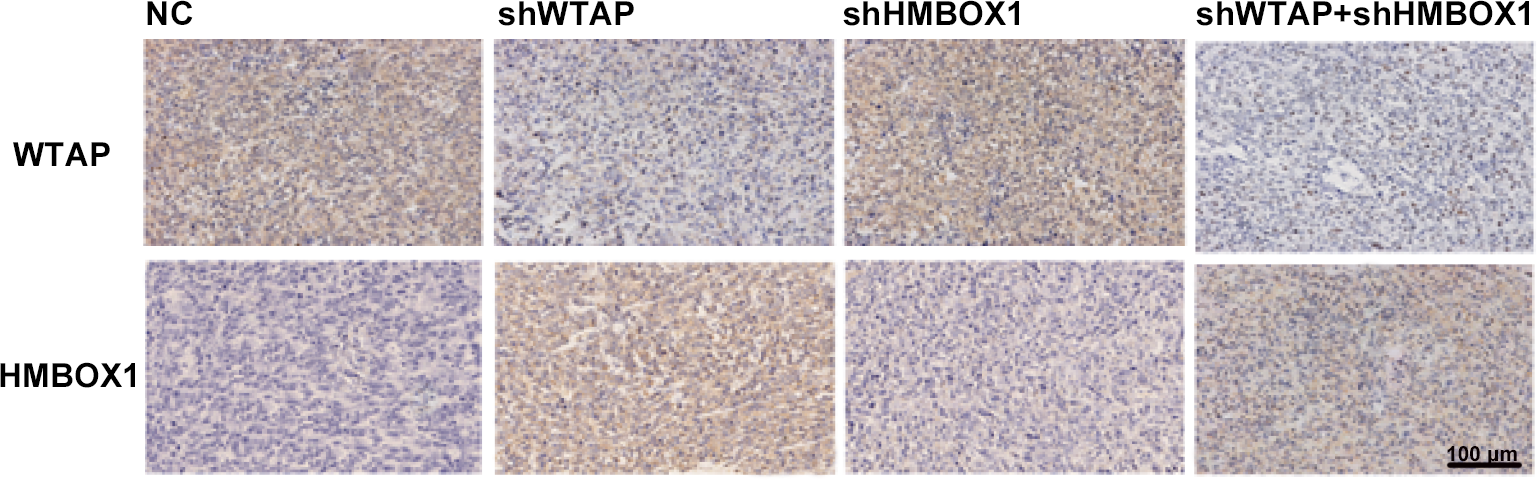

Supplement: Supplementary file 5 — Figure S5 [file 41419_2020_2847_MOESM5_ESM.tif]

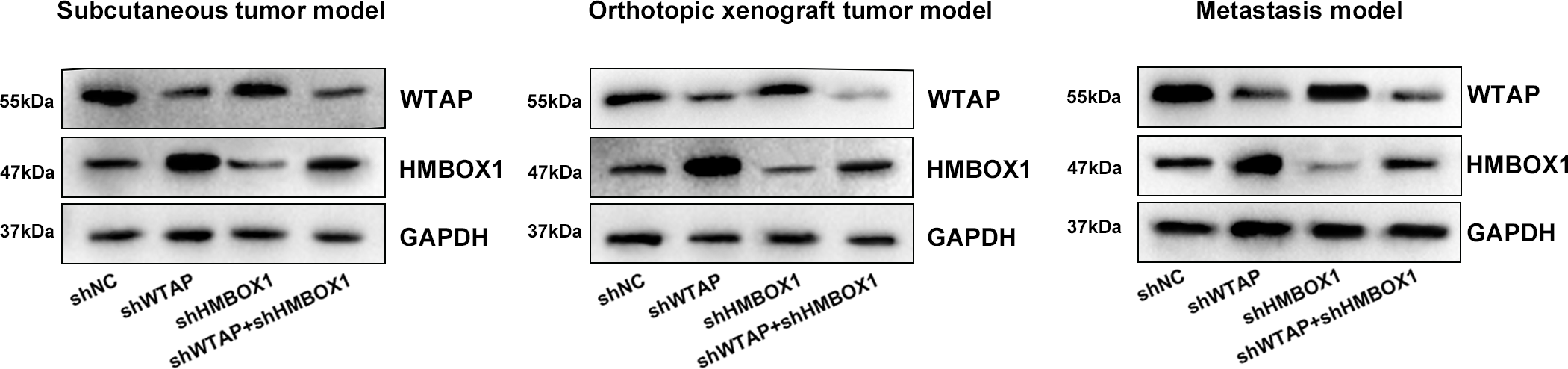

Supplement: Supplementary file 6 — Figure S6 [file 41419_2020_2847_MOESM6_ESM.tif]
