# Supplementary material for: WTAP promotes osteosarcoma tumorigenesis by repressing HMBOX1 expression in an m6A-dependent manner
Source: Cell Death Dis. 2020 Aug 19;11(8):659. doi: 10.1038/s41419-020-02847-6 (PMC7438489; doi:10.1038/s41419-020-02847-6)
Supplement: Supplementary file 7 — supplementary Figure Legends [file 41419_2020_2847_MOESM7_ESM.docx]

Supplementary Figure Legends

Figure S1. m6A-relative genes expression in OS tissue. A, 17 m6A-relative genes between osteosarcoma tissue and normal bone tissue (GSE87624: 3 normal and 44 osteosarcoma). B, WTAP expression in OS.

Figure S2. RNA-seq results revealed the DEGs between WTAP-silenced group and control sh-NC group. A, The volcano map and heatmap of DEGs. Red is the up-regulated genes, green is the down-regulated genes. B, GO pathway revealed the biological processes (BP), cellular components (CC), and molecular functions (MF) of DEGs. C, KEGG pathway and the network of enrichment pathway.

Figure S3. The expression of the overlapped genes in OS tissues. A, The expression of the overlapped genes in OS tissues from GSE87624. B, The expression of the overlapped genes in OS tissues from TXHCSU. C, The relationship between HMBOX1 expression and clinicopathological features in OS.

Figure S4. The high confidence m6A sites of HMBOX1 were predicted in SRAMP (http://www.cuilab.cn/sramp).

Figure S5. WTAP and HMBOX1 expression in OS tissue in mice.

Figure S6. WTAP and HMBOX1 expression in OS tissue in mice.

Table S1. The primer sequences for qPCR and shRNA.

| Gene symbol | Forward primer | Reverse primer |
| --- | --- | --- |
| GAPDH | GGAGCGAGATCCCTCCAAAAT | GGCTGTTGTCATACTTCTCATGG |
| WTAP | TTCCCAAGAAGGTTCGATTG | TGCAGACTCCTGCTGTTGTT |
| HMBOX1 | CTTCAGCGACTTCGGCGTA | ATCATAACTGTTGCTAGGTGACG |
| YTHDF2 | GCTTGCCTGCTACATAGTGAGA | AACTGAACTGCTTAACCTTCTGG |
| shWTAP#1 | CCGGGGGCAAGTACACAGATCTTAACCTCGAGGTTAAGATCTGTGTACTTGCCTTTTTG | AATTCAAAAAGGCAAGTACACAGATCTTAACCTCGAGGTTAAGATCTGTGTACTTGCC |
| shWTAP#2 | CCGGGGCAACACAACCGAAGATGACTCTCGAGAGTCATCTTCGGTTGTGTTGC TTTTTG | AATTCAAAAAGCAACACAACCGAAGATGACTCTCGAGAGTCATCTTCGGTTGTGTTGC |
| shHMBOX1#1 | CCGGG GGACCTAGATGTAGATGAT CTCGAGATCATCTACATCTAGGTCC TTTTTG | AATTCAAAAAGGACCTAGATGTAGATGATCTCGAGATCATCTACATCTAGGTCC |
| shYTHDF2#1 | GCCCAAUAAUGCAUAUACUTT |  |
| shYTHDF2#2 | GCUCUGGAUAUAGUAGCAATT |  |
